# An Eye Movement Desensitization and Reprocessing (EMDR) Group Intervention for Syrian Refugees With Post-traumatic Stress Symptoms: Results of a Randomized Controlled Trial

**DOI:** 10.3389/fpsyg.2018.00493

**Published:** 2018-06-12

**Authors:** Asena Yurtsever, Emre Konuk, Tuba Akyüz, Zeynep Zat, Feryal Tükel, Mustafa Çetinkaya, Canan Savran, Elan Shapiro

**Affiliations:** ^1^DBE Institute for Behavioral Studies, Istanbul, Turkey; ^2^BATE Individual and Family Therapy Institute, Istanbul, Turkey; ^3^Psychiatry Department, Medical School, Istanbul University, Istanbul, Turkey; ^4^Psychologist in Private Practice, Ramat Yishay, Israel

**Keywords:** EMDR, G-TEP, group therapy, refugee, PTSD, war, trauma

## Abstract

The number of refugees has increased significantly over the past few years. PTSD and depression are among the most common mental health problems among refugees. Eye Movement Desensitization and Reprocessing (EMDR), an effective treatment for PTSD, is usually administered individually. The availability of mental health resources would be greatly enhanced when EMDR can be delivered to groups. The EMDR G-TEP is a group protocol based on Early EMDR intervention protocols. There is clinical evidence and one field study published on the effect of EMDR G-TEP and there is only one RCT published on the treatment of PTSD and depression in a refugee camp. The aim of our study was to investigate the efficacy of EMDR G-TEP in treating post-trauma symptoms and depression and preventing the development of chronic PTSD among refugees living in a refugee camp. 47 adult participants with PTSD symptoms were randomly allocated to experimental (*n* = 18) and control (*n* = 29) groups. We measured Impact of Event Scale (IES-R), Beck Depression Inventory-II (BDI-II) and International Neuropsychiatric Interview (MINI) at pre-, post- and 4-week follow-up. Analysis of the results showed that the EMDR G-TEP group had significantly lower PTSD and depression symptoms after intervention. The percentage of PTSD diagnosis decreased from 100 to 38.9% in the EMDR G-TEP group and was unchanged in the control group. Following the EMDR G-TEP intervention 61.1% of the experimental group no longer had a PTSD diagnosis; this decrease was maintained at 4 weeks follow-up. In the control group the percentage of people who no longer met the diagnostic criteria for PTSD was 10.3% post-test and 6.9% at 4 weeks follow-up. A significant decrease in depression symptoms from pre-test levels was found in EMDR group but not in the control group follow up-test. This study indicated that EMDR G-TEP effectively reduced PTSD symptoms among refugees living in a camp, after two treatment sessions conducted over a period of 3 days. Further studies need to be performed using a larger number of participants, followed for a longer period of time and given more treatment sessions to strengthen our findings.

## Introduction

Over the last few years there has been a dramatic increase in the number of forcibly displaced people all around the world. The total number of refugees has increased significantly and consistently over the past 4 years. According to the UNHCR Mid-Year Trends 2015 Report, this number reached 59.5 million by the end of 2014 due to persecution, conflict, generalized violence, and human rights violations. This Report (2015) indicates that the number of refugees at the end of 2011 was 10.4 million and it had reached an estimated 15.1 million by mid-2015, its highest level in 20 years. The war in Syria has been the main contributor to this trend. Countries surrounding Syria have been heavily affected by this crisis. As one of these countries, Turkey hosts more than 2.6 million Syrian refugees (mid-February 2016, The UN Refugee Agency, 2016). By April, 2018 the total registered Syrian refugees number is 5,636,302 and 3,572,565 of which is in Turkey according to UNHCR. Given the large unregistered refugee population, the true figure may be even larger. The UNHCR Report also indicates that Turkey has the highest Syrian refugee number in the world.

Refugees have had to leave their homes because of various traumatic life experiences such as rape, torture, starvation, injury, and the threat of being murdered and the disappearance of family members. Research reveals that there is a strong relationship between mental health problems and the traumatic experiences in this population (Rousseau et al., 2001; Trautman et al., 2002). A study of refugees in camps on the Thailand-Cambodia border revealed that 55% of the population was diagnosed with depression while 15% of them had post-traumatic stress disorder (PTSD) (Mollica et al., 1993). EMDR has been used in cases of mass disaster (e.g. Jarero et al., 2006, 2008; Maxfield, 2008; Natha and Daiches, 2014; Allon, 2015; Maslovaric et al., 2017).

Moreover, it has been suggested that even in the absence of clinically significant symptoms, up to 68% of those who are exposed to traumatic life events are more likely to develop delayed onset PTSD (Andrews et al., 2007). North (2007) states that after a trauma people have various psychological problems including depressive reactions, phobias, alcohol and substance abuse, psychotic reactions and conversion symptoms. Likewise, Brady et al.'s study (Brady et al., 2000) conducted among assault victims demonstrated that following an adverse life event, victims might develop not only PTSD but also major depressive disorder (60%) and substance abuse (25%).

Mass traumas such as war, tsunami, and earthquake affect a significant number of people. The victims of trauma may have to face repeated exposure to stressors after the main event. They may face many difficulties including loss, migration and poverty, which they have to cope with as part of their daily life. These accumulated traumas can decrease their resilience and quality of life while increase the risk of health problems.

It is proposed that early intervention is important to prevent the development of more serious mental problems including PTSD, depression, anxiety, as well as to increase resilience and even to prevent conflict in community (Slobodin and de Jong, 2015). Since traumatic stress is a risk factor for PTSD and other trauma related disorders the need for an effective early intervention to treat distress and prevent the development of pathology is paramount.

The Cochrane reviews of controlled studies (Bisson and Andrew, 2007; Roberts et al., 2010) revealed that there are effective psychological interventions for people who are exposed to traumatic events. Many international clinical guidelines recommend Focused Cognitive Behavioral Therapy (CBT) and Eye Movement Desensitization and Reprocessing (EMDR Therapy) as treatments of choice for PTSD (e.g., Bisson and Andrew, 2007; World Health Organization, 2013; National Institute for Clinical Excellence, 2016).

EMDR as a brief, effective approach for processing traumatic memories is very suited for Early Intervention. EMDR Therapy is based on the Adaptive Information Processing (AIP) Model. Shapiro (2001), Shapiro and Solomon (1995), and Shapiro et al. (2007) states that “In terms of AIP current symptoms are viewed as resulting from disturbing experiences that have not been adequately processed and have been encoded in state-specific, dysfunctional form.” The heart of EMDR involves the transmutation of these dysfunctionally stored experiences into an adaptive resolution that promotes psychological health (Solomon and Shapiro, 2008).

Trauma can be conceptualized as an impairment of integrative functions. The intrusive fragmented elements of the traumatic memory cannot be assimilated and metabolized by the mind (Tofani and Wheeler, 2011). After the earthquake in the San Francisco Bay area in 1989, Francine Shapiro discovered that working with recent traumas required a different approach, since at some level of information processing the memory cannot have sufficient time to consolidate into an integrated whole. She proposed the Recent Event Protocol as an application of the standard EMDR protocol, conceptualizing the recent traumatic event as a fragmented experience that has not yet been consolidated and also reintroduced her original EMD Protocol for use in emergency situations (Shapiro, 2001). Based on these protocols, E. Shapiro and Laub developed the Recent Traumatic Episode Protocol (R-TEP) in 2008 (Shapiro and Laub, 2008). The EMDR R-TEP is an integrative recent trauma-focused protocol for Early EMDR Intervention (EEI). It includes procedures and measures for containment and safety. The intervention can be on consecutive days because no homework is required, The EMDR R-TEP protocol introduced a focus on the trauma *episode* rather than on only the initial trauma *event*. The original traumatic event, together with the traumatic aftermath, is seen as an ongoing *traumatic episode* continuum because the experiences are not yet consolidated, integrated or adaptively processed (see Jarero and Artigas, 2018). E. Shapiro later introduced a group application, the Group Traumatic Episode Protocol (G-TEP) in 2013 (Shapiro, 2013). It is adapted from EMDR Recent Traumatic Episode Protocol (R-TEP) for using with different age groups and populations who have experienced recent traumatic experiences or adverse events with ongoing impact not necessarily recent. The main goal is to use a group framework to process a Trauma Episode to reduce traumatic stress, promote adaptive processing, strengthen resilience and prevent post-trauma complications (Shapiro, 2015).

Considering the limited number of resources such as health care professionals, money, accommodation, time and the high number of refugees under the risk of post-traumatic stress, it is crucial to provide cost and time effective, easily learned and applied interventions. Therefore, we planned a study with Syrian refugees utilizing EMDR G-TEP. The aim of the study was to investigate the effectiveness of EMDR G-TEP Group Protocol to reduce trauma and depression symptoms and prevent the development of PTSD, among Syrian refugees living in a refugee camp. This was the third of a series of studies. The first was a pilot study that made minor changes to the EMDR Standard Protocol (Acarturk et al., 2015). The project included training and giving supervision to local therapists, working for the Ministry of Family and Social Policies and municipalities, in EMDR Level 1. We wanted to evaluate the effectiveness of our treatment of the refugees. A second pilot study utilizing the EMDR Recent Traumatic Episode Protocol (R-TEP) showed positive results, indicating that the implementation of the protocol significantly reduced PTSD and depression (Acarturk et al., 2016). As the pilot study appeared to be effective, the present study was conducted with a larger population.

## Materials and methods

### Design

This study was a single blind research comparing an experimental group, who received two sessions of EMDR G-TEP intervention, to a control group at three time points (pre-, post- and 4 weeks follow up test). Participants provided their written informed consent to participate in the study.

### Participants and procedure

**Figure 1:**
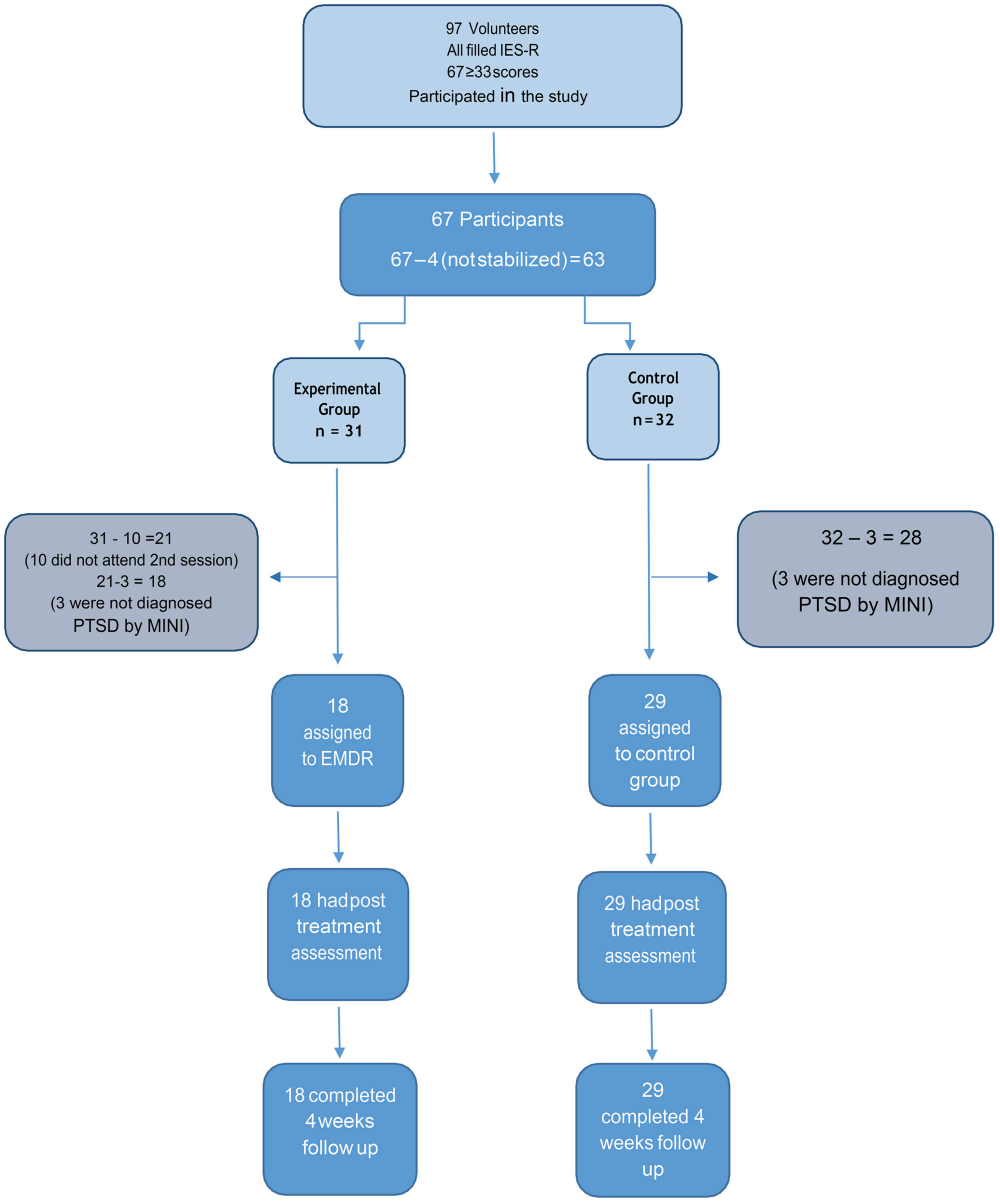
Study design and flow of patients throughout the trial.

This study took place at the Kilis Refugee camp in southeast Turkey on the Syrian border. Five therapists at the camp gave seminars about “war and trauma” at schools and leisure centers of the camp. The study and the therapy program was announced at several locations at the camp by the school and leisure center personnel in early September. Between September and October 2014 clinical staff at the Psychosocial Support Center within the camp identified potential participants who met the study inclusion criteria. Participants escaping from Syria due to war and taking refuge in Turkey, residing at the refugee camp, aged 18 and older and who had PTSD symptoms according to the IES-R (≥33) were invited to participate in the study. Ninety seven people intended to join the study. Participants who were pregnant, had mental retardation, psychotic, used psychiatric medication or were receiving any psychotherapy and refused to join the study were excluded. The number of participants who enrolled in the study was 67 (Figure 1), but four participants had to be excluded because they could not manage self—containment during the screening part of EMDR G-TEP. Participants who had an IES-R score of equal or above 33 were randomly assigned by a computer program to the experimental group (EMDR G-TEP = 31) and the EMDR control group (control group = 32). Ten people from the experimental group were unable to attend two sessions of G-TEP and so were also excluded from the study (*n* = 21). The demographic and pre-test characterisitcs of these ten subjects was similar to those who completed the two sessions. MINI test was applied to participants. The result showed that three participants in both experimental and control group were not diagnosed with PTSD so they were excluded from the study. The remaining 47 participants were randomly assigned to groups (experimental group = 18 and control group = 29, see Figure 1). As there was a common prejudice about getting psychological help, especially among men, the number of male participants were small (*n* = 12; 19%). It is recognized that conducting quality research in emergency situations has inherent difficulties and is likely to require some compromises with gold standard guidelines (Yehuda et al., 2015; Shapiro et al. submitted 2018).

### Measurements

There were three instruments used in this research:

Beck Depression Inventory-II (BDI-II): A 21-question self- report inventory, for depression (Beck et al., 1996). The Arabic version of BDI-II was developed by Ghareeb (2000) using 17 different Arabic speaking populations including Syrians. The total BDI score varies between 0 and 63, and a score of 11–16 indicates: Depressive Mood, 17–20: Mild Clinical Depression, 21–30: Moderate Depression 31–40: Severe Depression and 40 and above: Very Severe Depression.

Impact of Events Scale (IES-R): The Impact of Event Scale—Revised (IES-R), has 22 questions, 5 of which were added to the original IES to better capture the DSM-IV criteria for PTSD (Weiss and Marmar, 1997). The validity of IES-R has been tested in different populations (Panahi et al., 2011). Based on previous studies, we used a cutoff score of ≥33 as indicating the presence of PTSD (Weiss and Marmar, 1997). The scale was translated to Arabic by two independent translators. After back translation the scale yielded a Cronbach's alpha of α = 0.93 (Zaghrout, unpublished manuscript). Moreover, previous research with Syrian refugees indicated good psychometric properties of the scale (Acarturk et al., 2015).

MINI International Neuropsychiatric Interview (MINI): The Mini International Neuropsychiatric Interview (MINI) is a short diagnostic structured interview, developed in clinician (MINI-CR) and patient-rated (MINI-PR) formats, with 17 Diagnostic and Statistical Manual (DSM)-III-R Axis I psychiatric disorders (Sheehan et al., 1998). The Arabic version was developed by Kadri et al., in Moroccan Arabic in (Kadri et al., 2005).

The camp residents who were interested in participating in this study were screened based on the eligibility criteria mentioned above. The testers who spoke Arabic and Turkish fluently applied the instruments to the volunteers. Then, a final list was formed among applicants over 18 years old who had PTSD measured with the MINI and an IES-R score with the cutoff point ≥33 (Creamer and Falilla, 2002). The experimental and control groups were frequency matched for gender, age, marital status and education.

## EMDR G-TEP group intervention

The experimental group participants received two sessions of EMDR G-TEP in total, on three consecutive days. The group sessions took approximately 4 hours because the translation during the session doubled the time. Moreover, the participants needed breaks. The psychometric measures were applied to both experimental and control groups before the EMDR G-TEP group therapy started, a week after treatment and then a month later. None of the therapists who ran the groups took a role in conducting the surveys of the participants or saw the results. The EMDR G-TEP team consisted of four professionals who had EMDR Level 1 and Level 2 training and experience in EMDR of 3–15 years. The team received the EMDR G-TEP training from Elan Shapiro, the originator of the protocol, in 2013 and used it with different populations in order to prepare for the study.

### Statistical methods

In order to test whether there were statistically significant differences between the experimental and control groups in the categorical sociodemographic characteristics, chi-square or Fisher's exact test where appropriate were performed. Independent sample *t*-test was used to test whether there was a difference in age, IES-R and BDI-II between the two groups. In order to determine whether there were group differences in IES-R and BDI-II, a two factor Repeated measures ANOVA was used for pre-, post- and follow up tests. One way Repeated measures ANOVA was also used for pre-, post- and follow up tests. Bonferroni *post-hoc* testing was used to analyze the differences between the experimental and control group post and follow-up scores. In order to test whether there were differences in the percentage of participants with PTSD between groups, chi-square tests were conducted at each time point. In order to test whether there was a reduction in the percentage of PTSD among the participants over time, chi- square tests were conducted within each group. Significance was considered to be *p* < 0.05. Statistical analysis was performed using Statistical Package for the Social Sciences (SPSS) for Windows 19.0.

### Results

There was no statistically significant difference between the experimental and control group in sociodemographic variables (see Table 1). In addition, there was no statistically significant difference in age [experimental group: 39.89, control group: 35.93, *t*_(45)_ = 1.196; *p* > 0.05].

**Table 1:** Demographic characteristics of groups at baseline.

**Characteristic**	**Total (*n* = 47)**	**Experimental (*n* = 18)**	**Control (*n* = 29)**	**Analysis**		
				**χ^2^**	***df***	***p***
Gender				0.311	1	0.577
Male	11(23.4%)	5(27.8%)	6(20.7%)			
Female	36(76.6%)	13(72.2%)	23(79.3%)			
Marital status				0.038	2	0.981
Married	39(83.0%)	15(83.3%)	24(82.8%)			
Single	3(6.4%)	1(5.6%)	2(6.9%)			
Divorce	5(10.6%)	2(11.1%)	3(10.3%)			
Education				6.83	3	0.078
Not reading	6(12.8%)	3(16.7%)	3(10.3%)			
Primary School	25(53.2%)	6(33.3%)	19(65.5%)			
Middle School	6(12.8%)	2(11.1%)	4(13.8%)			
High School/University	10 (21.3%)	7(38.9%)	3(10.3%)			
Mean age	37.45(11.08)	39.89(10.96)	35.93(11.1)	*t* = 1.196	45	0.238
IES-R	62.45(11.04)	62.44(9.05)	62.45(12.2)	*t* = −0.001	45	0.999
BDI-II	31.85(10.99)	35.83(14.55)	29.38(7.3)	*t* = 1.97	45	0.051

*EMDR, Eye movement desensitization and reprocessing; df, degrees of freedom; S.D., standard deviation; BDI- II, Beck Depression Inventory-II; IES-R, Impact of Event Scale—Revised*.

In this study a 2 (groups) × 3 (time) factor RM Anova was used to evaluate the IES-R and BDI measures (see Table **3**). The procedure was run on 47 patients representing experimental and control groups.

Pre-treatment mean IES-R was 62.44 (see Table 2). Repeated measures analysis revealed no significant group effect [*F*_(1, 45)_ = 3.07; *p* > 0.05, effect size = 0.064]. In addition, there was a significant time effect [*F*_(2, 90)_ = 6.46; *p* < 0.01, effect size = 0.126]. Group by time interaction was not significant [*F*_(2, 90)_ = 2.26; *p* > 0.05, effect size = 0.048].

**Table 2:** Means (standard deviations) of the two measures over time.

	**Pre**		**Post**		**Follow**	
	**EMDR**	**Control**	**EMDR**	**Control**	**EMDR**	**Control**
**MEASURE**						
IES-R	62.44 (9.05)	62.45 (12.27)	48.22 (17.34)	59.10 (17.37)	51.94 (16.78)	58.83 (15.41)
BDI-II	35.83 (14.55)	29.38 (7.32)	28.00 (9.75)	26.10 (10.98)	24.67 (12.59)	24.41 (11.61)

Bonferroni *Post-hoc* testing of the time effect revealed a statistically significant difference between the pre-test and post-test scores of IES-R (difference = 8.78, se = 2.77; *p* < 0.01) as well as a statistically significant difference between the pre-test and follow-up scores of IES-R (difference = 7.06, se = 2.19; *p* < 0.01). There was no significant difference between the mean post- test and follow-up scores of IES-R (difference = −1.72, s e = 2.77; *p* > 0.05).

As we were particularly interested in the treatment effect, a RM ANOVA was performed on the experimental group (Table 3). We found meaningful results (F *P* < 0.05). For this reason we used *post-hoc* tests. The same procedure was used for the control group.

**Table 3:** 2 × 3 Repeated ANOVA results for IES-R and BD-II.

	**Group**			**Time**			**Time** × **Group**		
	***df***	***F***	**η2**	***df***	***F***	**η2**	***Df***	***F***	**η2**
**MEASURE**									
IES-R	1.45	3.06^*^	0.064	2.90	6.46^***^	0.126	2.90	2.26	0.048
BDI-II	1.45	1.278	0.028	2.90	9.86^***^	0.180	2.90	1.493	0.032

* p < 0.05;

*** *p < 0.001*.

Bonferroni *Post-hoc* testing of the time effect for the experimental group revealed a statistically significant difference between the pre-test and post-test scores of IES-R (difference = 14.22, se = 4.81; *p* < 6.05) as well as a statistically significant difference between the pre-test and follow- up scores of IES-R (difference = 10.5, se = 4.10; *p* < 0.05). There was no significant difference between the mean post-test and follow-up scores of IES-R (difference = −3.72, se = 3.76; *p* > 0.05).

There was no significant difference in the control group's pre-test and post-test (difference = −3.35, se = 3.18; *p* > 0.05), pre-test and the follow-up (difference = 3.62, se = 2.35; *p* > 0.05) and post- test and the follow-up IES- R mean scores (difference = −0.28, se = 3.67; *p* > 0.05).

Independent sample *t*-test of the two groups at each time point revealed that there was no statistically significant difference between the IES-R pre-test scores between the two groups (*t* = −0.001, *df* = 45; *p* > 0.05). At post-test, the experimental group had a significantly lower mean score as compared to the control group (*t* = −2.09, *df* = 45; *p* < 0.05). But at the follow-up test there was no statistically significant difference between the experimental group and the control group (*t* = −1.439, *df* = 45; *p* > 0.05).

Pre-treatment mean BDI was 31,85 indicative of severe depression. Repeated measures analysis revealed that there was no significant group effect [*F*_(1, 45)_ = 1.28; *p* > 0.05, effect size = 0.028]. However, there was a significant time effect [*F*_(2, 90)_ = 9.86; *p* < 0.001, effect size = 0.180]. In addition, there was no significant difference between Time × Group interaction [*F*_(2, 90)_ = 1.49; *p* > 0.05, effect size = 0.032].

*Post-hoc* testing revealed that there was a significant difference between the mean pre-test and follow-up scores of BDI-II (difference = 5.56, se = 1.78; *p* < 0.01). As well as a statistically significant difference between the pre-test and follow-up scores of IES-R (difference = 8.07, se = 1.96; *p* < 0.001). There was no significant difference between the mean post-test and follow-up scores of BDI-II (difference = 2.51, se = 1.84; p > 0.05).

RM of the experimental group revealed a significant difference between the mean pre-test and post-test scores of BDI-II (difference = 7.83, se = 2.84; *p* < 0.05) as well as a statistically significant difference between the pre-test and follow-up scores of BDI-II (difference = 11.17, se = 3.28; *p* < 0.01). There was no significant difference between the mean post-test and follow-up scores of BDI-II (difference = 3.33, se = 2.23; *p* > 0.05).

On the other hand, there was no significant difference in the control group's pre-test and post-test (difference = 3.28, se = 2.08; *p* > 0.05), pre-test and the follow-up (difference = 4.97, se = 2.32; *p* > 0.05) and post- test and the follow-up BDI-II mean scores (difference = 1.69, se = 2.53; *p* > 0.05).

The statistical analysis done for MINI scale (see Table 4) for each group separately revealed a time effect in the experimental group (χ2 = 14.8, *p* < 0.001) but not in the control group (χ2 = 2.80, *p* > 0.05). Time effect within the experimental group revealed a significant decline in the percentage of participants with PTSD between pre-test (100.0%) and both post- (44.4%; *p* < 0.01) and follow-up (38.9%, *p* < 0.01). There was no statistically significant difference in the percentage of PTSD between post and follow-up test times (*p* > 0.05).

**Table 4:** PTSD diagnosis according to the MINI assessment.

**MINI**		**Pre**		**Post**		**Follow up**		
**Group**	**Yes**	**No**	**Yes**	**No**	**Yes**	**No**	**_χ_2**	***P***
Experimental	18	0	8	10	7	11	14.8	0.000^**^
	(100.0%)	(0.0%)	(44.4%)	(55.6%)	(38.9%)	(61.1%)		
Control	29	0	26	3	27	2	2.80	0.250
	(100.0%)	(0.0%)	(89.7%)	(10.3%)	(93.1%)	(6.90%)		
χ^2^	0.000		11.35		16.32			
p	>0.05		<0.001		<0.001			

*Data is N (%)*.

** p < 0.01

There was no decrease in the trauma symptoms in the Control group. Following the EMDR G-TEP intervention post-test results demonstrated that 55.6% of the experimental group after 2 days of EMDR Therapy and 61.1% of the experimental group at the follow up no longer had a PTSD diagnosis.

## Discussion

To our knowledge this is the first study performed to evaluate the effectiveness of the EMDR G-TEP Group Protocol (a later study has subsequently been published (Lehnung et al., 2017) and the third RCT conducted in a refugee camp setting. (The first and second RCTs were conducted by Acarturk et al. (2015, 2016).

The aim of this study was to examine the effectiveness of the EMDR G-TEP Group Protocol as an early intervention to reduce the PTSD diagnosis compared to the control group, the trauma symptoms and depression and prevent the development of PTSD among refugees living in a camp. As expected, after the EMDR G-TEP 61% of the clients did not receive PTSD diagnosis at the follow up any more, whereas the control group remain the same.

As mentioned earlier, the total IES-R score of trauma symptoms in the EMDR G-TEP group decreased significantly and the effects were maintained a month later. The post-test mean score for IES-R post-trauma symptoms was significantly less than the control group mean score. At the follow-up test there was no statistically significant difference between the experimental group and the control group.

The same result applies for the BDI scores. In line with the reduction of trauma symptoms, the percentage of PTSD diagnosis in the EMDR G-TEP group decreased significantly. The depression scores of the EMDR G-TEP group decreased significantly (diff = 7.83) and there was no significant difference in the control group's pre-test and post-test (diff = 3.23).

In this study we expected that the follow up test scores would be different too, but there was no difference between the experimental and control groups. We may explain this with the unusual circumstances and life going on in the refugee camps. After the treatment the experimental and the control groups continued their life at the camp Situations that the participants had to face each day in the camp, which is located close to the border, exposed them to ongoing stress as they were constantly triggered by re-traumatizing news about the war (e.g., violence; tortures, rapes, mass murders etc.,). They watched the TV channels where there were violent killings of their citizens, their husbands, wives and sons, who were fighting in Syria. That is their traumas continued being triggered, and may be new traumas have been developed. That is why we used G-TEP twice. It seems two sessions were not enough to reduce the scores more than the scores of the control group. If we regard this as a pilot study, in the future trials, we may do G-TEP three times or more.

The results of our study suggest that a group intervention with the EMDR G-TEP protocol can be used effectively with adults as an intervention during a period of significant on-going disruption and trauma, for screening and reducing symptoms of post-traumatic stress, self- reported distress and possibly for the reduction of depression.

Our study showed that EMDR G-TEP is an efficient group model, in terms of time, cost and resources, even in a situation of ongoing crisis, violence and war conditions with the effects maintained. A review of the literature showed that there are very few controlled studies on early interventions after large scale disasters. The research about refugees in a camp setting is even less studied. Therefore, this study stands out in this field. Further studies need to be done with different populations.

### Limitations and lessons

Due to practical and logistic difficulties we could only conduct the study with a relatively small number of participants over a limited time period. Also, the absence of a long term assessment of the control group is another limitation of the study.

It should be noted that for ethical considerations, after conducting EMDR G-TEP with the intervention group, we intended to complete EMDR G-TEP treatment with the control group as well (delayed treatment). However, due to unfortunate bureaucratic and security circumstances our request to continue and complete EMDR G-TEP with the control group was not possible. Therefore, EMDR therapists at the camp offered individual EMDR therapy to the control group and one third of them received EMDR Therapy.

Although the EMDR G-TEP can be conducted by a single therapist, considering the severity of the trauma in this population, with a possibility of intense abreactions and dissociation we decided to work with two therapists in each group. This gave us the opportunity to intervene one-on one if necessary. Finally, the worksheet format assumed the participants to be literate and to be able to follow the instructions. However, some of our participants were illiterate and they needed extra assistance. The option of using drawings as well as written expression here was helpful in this regard, but it should be taken into consideration in future studies. These aspects can be aided by employing paraprofessional support staff alongside the therapists.

## Ethics statement

EMDR Turkey Association Research Committee: The permission to get into the refugee camp was given for a short period and in an unexpected time so we were not able to get the ethical committee of the regional university. Therefore, the ethical approval was given by the EMDR Turkey Research Committee. The study was announced in the refugee camps by the leaders of the tribes in the camp. They explain the aim of the project to their members and were asked to be applied. Before the sessions begin, the team members gave a short information about the trauma and they were told that the aim of the study was to reduce the effect of the trauma that they were going through. Their names were written on a paper and they signed their confirmation to participate in the study. They were also told that if they feel the process is too difficult to carry on they can always leave the study. They were also assured that they will have an individual EMDR Therapy after the group work until they feel comfortable.

## Author contributions

All authors listed have made a substantial, direct and intellectual contribution to the work, and approved it for publication.

### Conflict of interest statement

The authors declare that the research was conducted in the absence of any commercial or financial relationships that could be construed as a potential conflict of interest.
